# DAS181 Treatment of Severe Parainfluenza Virus 3 Pneumonia in Allogeneic Hematopoietic Stem Cell Transplant Recipients Requiring Mechanical Ventilation

**DOI:** 10.1155/2016/8503275

**Published:** 2016-01-28

**Authors:** B. Dhakal, A. D'Souza, M. Pasquini, W. Saber, T. S. Fenske, R. B. Moss, W. R. Drobyski, P. Hari, M. Z. Abidi

**Affiliations:** ^1^Division of Hematology Oncology, Department of Medicine, Medical College of Wisconsin, Milwaukee, WI 53226, USA; ^2^Ansun BioPharma, San Diego, CA 92121, USA; ^3^Division of Infectious Disease, Department of Medicine, Medical College of Wisconsin, Milwaukee, WI 53226, USA

## Abstract

Parainfluenza virus (PIV) may cause life-threatening pneumonia in allogeneic hematopoietic stem cell transplant (HSCT) recipients. Currently, there are no proven effective therapies. We report the use of inhaled DAS181, a novel sialidase fusion protein, for treatment of PIV type 3 pneumonia in two allogeneic hematopoietic SCT recipients with respiratory failure.

## 1. Introduction

In allogeneic hematopoietic stem cell transplant (HSCT) recipients, parainfluenza (PIV) virus can cause varying spectrum of respiratory illness, ranging from self-limited upper respiratory tract infections (URTI) to severe, life-threatening lower respiratory tract infections (LRTI) [1, 2]. In HSCT recipients and patients with hematologic malignancies, progression from URTI to LRTI is estimated to occur in 13% to 43% of cases, whereas mortality rates from PIV LRTI are reported between 12% and 50% [3]. Clinical manifestations may vary in severity based upon the type of transplant and immune suppression [4]. The mainstay of PIV treatment in HSCT recipients remains to be largely supportive care along with reduction of immune suppression, as currently there is a lack of antiviral agents or vaccines with proven efficacy [5]. DAS181 is a novel investigational sialidase fusion protein, which removes sialic acid-containing receptors from the surface of respiratory epithelial cells, and thus prevents PIV and influenza virus from binding to these cells [6]. DAS181 has been shown to be safe in phase I and II clinical trials for treatment of influenza [7] and also has also been shown to have* in vitro* and* in vivo* activity against PIV [8, 9]. In recent years, there have been a very limited number of HSCT and lung transplant recipients with PIV LRTI treated with DAS181 with improvement in symptoms, viral loads, and oxygen requirements [10–14]. We describe use of DAS181 in two critically ill allogeneic HSCT recipients who presented with PIV type 3 (PIV3) LRTI.

## 2. Methods

Patients were diagnosed with PIV3 infection by viral nucleic acid amplification test (NAAT) done on nasopharyngeal swab and bronchoalveolar fluid lavage (BAL) samples. In each case, DAS181 (Ansun BioPharma, Inc., San Diego, California) was obtained through an emergency investigational new drug (EIND) application and was approved by the US Food and Drug Administration and the Medical College of Wisconsin Institutional Review Board. Informed consent was obtained from the patients' families. DAS181 was administered as a nebulized solution for 5–10 days. The appropriate dose was then administered over 10 minutes, based on manufacturer recommendations. Daily assessment was carried out for clinical and laboratory parameters and for any adverse events. The following laboratory tests were monitored while patients received treatment: complete blood count with differential, electrolytes, alkaline phosphatase, alanine aminotransferase, and aspartate aminotransferase. After completion of therapy complete blood counts and liver function tests were monitored on a weekly basis after completion of therapy.

**Case 1 .**

The patient was a 74-year-old female with myelodysplastic syndrome (MDS) who was treated with hypomethylating agents and then underwent a 7/8 HLA-mismatched related peripheral blood allogeneic SCT in April 2015 using reduced-intensity conditioning with melphalan, fludarabine, and 200 cGY total body irradiation. She was on mycophenolate mofetil and tacrolimus for graft versus host disease (GVHD) prophylaxis. After transplant, she remained pancytopenic secondary to chemotherapy. On transplant day (TxD) + 18, the patient developed a new productive cough, fevers and was noted to be more tachypneic. She was found to be positive for PIV3 by viral NAAT on nasopharyngeal swab. Intravenous immune globulins (IVIG) and corticosteroid therapy were administered; however her respiratory status continued to worsen, requiring transfer to the intensive care unit for intubation on TxD + 24. Bronchoscopy with bronchoalveolar lavage (BAL) was performed and the NAAT for PIV3 was positive on the BAL fluid. DAS181 was obtained by EIND and was administered via Aeroneb nebulizer starting on TxD + 25. At onset of therapy, decision was made to limit treatment course of DAS181 for five days. The only appreciable side effect of DAS181 was an increase in alkaline phosphatase (ALK), which peaked at 288 (35–104 units/Liter) at day 4 of therapy. Baseline ALK prior to start of therapy was 177 units/Liter. At the end of the 5-day course of DAS181, while she remained intubated, her oxygen requirements had improved. Her fraction of inspired oxygen (FiO2) requirements decreased to 40%, positive end expiratory pressure (PEEP) 5 with oxygen saturation of 100%. She subsequently displayed signs of sepsis, which was attributed to intravascular catheter infection with* Staphylococcus epidermidis* and was treated with IV linezolid. Despite receipt of growth factor she failed to have hematopoeitic recovery. Her fevers persisted with increased pressor requirements and she developed multiorgan failure. The decision was made by her family to withdraw care. She expired on day TxD + 32.

**Case 2 .**

A 35-year-old Caucasian woman presented with cough and shortness of breath at day TxD + 324 after myeloablative allogeneic matched unrelated donor peripheral blood SCT for acute myeloid leukemia in second complete remission. After transplant, she developed acute GVHD involving the skin and gut and subsequently chronic GVHD involving the skin and oral cavity as well as upper gastrointestinal tract in the form of eosinophilic esophagitis. Her ongoing immunosuppression regimen included corticosteroids, mycophenolate mofetil, and extracorporeal photopheresis. Computerized tomography (CT) scan of the chest showed new diffuse nodular infiltrates with multifocal consolidation bilaterally. Urine pneumococcal antigen was positive and she was started on intravenous (IV) antibiotics. A viral NAAT done on a nasopharyngeal swab was positive for PIV3, and the patient received oral ribavirin for 7 days. On hospital day 4, she was discharged home on oral antibiotics and ribavirin. She was readmitted a week later, with worsening cough, dyspnea, and increased oxygen requirements and was empirically started on IV broad-spectrum antibiotics and underwent bronchoscopy with BAL. The BAL fluid showed PIV3 by NAAT, evidence of cytomegalovirus viral DNA, and 1,000 colony forming units/mL of* Pseudomonas aeruginosa*. Although blood CMV NAAT remained negative, she was started on IV ganciclovir and IVIG for possible CMV pneumonitis. She continued to worsen and required mechanical ventilation. On hospital day 6, DAS181 use was obtained and administered via an Aeroneb nebulizer. She received 10 days of therapy with DAS181 and we observed a decrease in her oxygen requirements during the course of therapy. Only side effect was an asymptomatic increase in ALK levels, which peaked at 261 (35–104 units/Liter) by day 6 of therapy. Prior to start of therapy, ALK was 88 units/Liter. This resolved spontaneously after completion of therapy. Her ICU stay was complicated by the development of* Pseudomonas aeruginosa* ventilator associated pneumonia on hospital day 21, which was successfully treated. A marked clinical and radiologic improvement was seen and she was discharged from the ICU and eventually extubated after a tracheostomy and a total of thirty days of mechanical ventilation.

## 3. Discussion

PIV infections cause substantial morbidity and mortality in SCT recipients and have no effective treatment [5, 15]. Care is mostly supportive and includes supplemental oxygen, treatment of fungal and bacterial coinfections and reduction of immune suppression. Neither IVIG nor inhaled ribavirin have shown to reduce duration of PIV shedding or PIV-related mortality in SCT recipients [5]. In a recent study, Seo et al. described ribavirin use to be associated with lowering of overall mortality [16]. However, this study failed to show any benefit in deaths due to respiratory failure or in patients with BAL confirmed LRTI. Other studies have consistently failed to show efficacy of ribavirin in HSCT recipients [1, 5, 15].

DAS181 (Ansun BioPharma, Inc., San Diego, California) is a novel sialidase fusion protein that has shown* in vitro* efficacy against PIV in a cotton rat infection model as well as in lung transplant and HSCT recipients [8, 10–14]. Compared with previously published cases of use of DAS181 for PIV3 in adult HSCT recipients (Table 1), Case 1 presented very early after transplant and failed to engraft despite growth factor stimulation which played a significant role in her early demise despite receipt of a 5-day course of DAS181.

While optimal treatment duration is unknown, it is possible that a longer treatment duration prevented recurrence of infection in Case 2. In previously reported cases in HSCT recipients, a 5-day course of nebulized DAS181 was successful in clearing the infection in two patients with severe PIV LRTI requiring mechanical ventilation [10]. However, one of these patients developed recurrence of PIV and died. The authors suggested that a longer treatment course might reduce risk of relapse of PIV in these critically ill patients [10, 11].

The adverse effects of DAS181 treatment were mild and self-limited in our patients and did not impact duration of treatment. Transient elevations in alkaline phosphatase were seen in both cases as described in the literature.

Limitations of our study include description of only two critically ill allogeneic HSCT recipients who received DAS181. Our data would have been strengthened with serial viral load studies. In the absence of this information, we are unable to unequivocally attribute the role of DAS181 in viral clearance.

## Figures and Tables

**Table 1:** Summary of cases of DAS181 treatment for PIV3 pneumonia in SCT recipients described in the literature.

	Case 1	Case 2	Case 3	Case 4	Case 5	Case 6	Case 7
Age (y)/sex	63/F	64/F	69/M	12/M	7 months/M	4/M	3/M

Underlying disease	AML	Unknown	MDS	Relapsed ALL	SCID	Stage 4 neuroblastoma	AML

Type of transplant	Reduced intensity Allogeneic HSCT from MUD	Nonmyeloablative Allogeneic HSCT	Allogenic HSCT	Double cord	Single cord	Autologous HSCT	MUD peripheral blood HSCT

GVHD	Day 24	Chronic	Chronic	None	None	None	None

Immune suppression for GVHD	Corticosteroids Mycophenolate mofetil	Prednisone Sirolimus	Prednisone	None	None	None	None

Onset of PIV							
URTI	URTI: TxD + 89			URTI: TxD + 260	URTI: TxD + 71	URTI: TxD + 26	URTI: TxD + 2
LRTI	LRTI: TxD + 124	6 y SP SCT	LRTI: TxD + 240	LRTI: TxD + 275	LRTI: TxD + 2	LRTI: TxD + 25	

Type of PIV	PIV3	PIV1	PIV3	PIV3	PIV2	PIV3	PIV3

Radiographic findings	Tree in bud opacities, mild diffuse bronchial thickening consistent with viral bronchiolitis on CT	Ground glass opacities on CT	Ground glass opacities on CT	Ground glass opacities on CXR/CT	Infiltrates on CXR/CT	New infiltrates on CXR/CT	

Clinical symptoms	Cough, dyspnea, increased oxygen requirements	Respiratory distress, increased oxygen requirements	Fever, hemoptysis, and respiratory distress	Respiratory distress, increased oxygen requirements	Fever, wheezing, and increased oxygen requirements	Fever, cough, and crackles	Cough, rhinorrhea

Copathogens	Reactivated CMV viremia (prior to PIV URTI)	None	None	Adenovirus and hMPV (at time of URTI) HRV and Adenovirus (at time of LRTI)	None	None	HRV

Mechanical ventilation	None	Intubated	Intubated	None	None	None	None

DAS181 treatment	10 mg/day × 3 days (dry inhaled powder)	3.2 mg/day × 3 days (nebulized) and then 4.5 mg/day × 2 days (nebulized and face mask)	3.2 mg/day × 3 days and then 4.5 mg/day × 2 days (nebulized)	10 mg/day × 10 days (dry powder)	0.14 mg/kg per day × 2 days and then 0.2 mg/kg per day × 3 days (nebulized)	0.14 mg/kg per day × 2 days and then 0.2 mg/kg per day × 3 days (nebulized)	0.14 mg/kg per day × 10 days (nebulized)

Maximum alkaline phosphatase while on treatment, IU/L (normal range)	Not reported	240 (35–104)	169 (35–104)	477 (135–530)	298 (95–380)	598 (95–380)	247 (95–380)

Outcome	Died of relapsed AML	Died	Alive	Alive	Alive	Alive	Alive

Year/reference	2011 [11]	2014 [10]	2014 [10]	2015 [14]	2015 [14]	2015 [14]	2015 [14]

AML, acute myeloid leukemia; MDS, myelodysplastic syndrome; ALL, acute lymphoblastic leukemia; SCID, severe combined immunodeficiency; MURD, matched unrelated donor; URTI, upper respiratory tract infection; LRTI, lower respiratory tract infection; TxD, transplant day; CXR chest Xray; CT computerized tomography; CMV, cytomegalovirus; hMPV, human metapneumovirus; HRV, human rhinovirus.
